# Distinguishing the milk microbiota of healthy goats and goats diagnosed with subclinical mastitis, clinical mastitis, and gangrenous mastitis

**DOI:** 10.3389/fmicb.2022.918706

**Published:** 2022-08-25

**Authors:** Richard Costa Polveiro, Pedro Marcus Pereira Vidigal, Tiago Antônio de Oliveira Mendes, Ricardo Seiti Yamatogi, Luciana Saraiva da Silva, Juliana Miwa Fujikura, Mateus Matiuzzi Da Costa, Maria Aparecida Scatamburlo Moreira

**Affiliations:** ^1^Laboratório de Doenças Bacterianas, Setor de Medicina Veterinária Preventiva e Saúde Pública, Departamento de Veterinária, Universidade Federal de Viçosa, Viçosa, MG, Brazil; ^2^Núcleo de Análise de Biomoléculas (NuBioMol), Centro de Ciências Biológicas, Universidade Federal de Viçosa, Viçosa, Minas Gerais, Brazil; ^3^Departamento de Bioquímica e Biologia Molecular, Universidade Federal de Viçosa, Viçosa, Minas Gerais, Brazil; ^4^Faculdade de Medicina, Universidade Federal de Uberlândia, Uberlândia, Minas Gerais, Brazil; ^5^Departamento de Zootecnia, Universidade Federal do Vale do São Francisco, Petrolina, Pernambuco, Brazil

**Keywords:** microbiota, goat milk, mastitis, bacteria, metataxonomic

## Abstract

Mastitis, mainly caused by bacterial intramammary infections, is the main problem in the breeding of dairy animals. The inflammations of the mammary gland is separated by types of mastitis, being subclinical, clinical, and the most severe, gangrenous mastitis. Here, we used 16S rRNA amplicon sequencing to characterize the bacterial microbiota of goat milk in the different types of goat mastitis caused by bacteria. We used 72 goat milk samples from a region of the state of Minas Gerais in Brazil, of which 12 were from clinically healthy animals, 42 from animals diagnosed with subclinical mastitis, 16 from animals with clinical mastitis, and 2 from animals with gangrenous mastitis. The group related to gangrenous mastitis was the most divergent in terms of alpha and beta diversity. The most abundant genus among samples of the groups was *Staphylococcus* spp., and we found a high abundance of *Mycoplasma* sp. in the milk of animals diagnosed with clinical mastitis. The most statistically relevant microorganisms among the groups were *Prevotella* sp., *Ruminococcaceae*, *Prevotella ruminicola* sp., and *Providencia* sp. We highlight a new association of bacterial agents in gangrenous mastitis among *Escherichia* sp./*Shigella* sp. and *Enterococcus* sp. and provide the second report of the genus *Alkalibacterium* sp., in milk samples. Only the taxa *Staphylococcus* sp., *Bacteroides* sp., *Enterococcus*, and *Brevidabacterium* sp., were present in all groups. The superpathway of L-tryptophan biosynthesis metabolites and the sucrose degradation III (sucrose invertase) pathway were the most prominent ones among the groups. In this study, we demonstrate how a rich microbiota of goat milk from healthy animals can be altered during the aggravation of different types of mastitis, in addition to demonstrating new bacterial genera in milk not previously detected in other studies as well as new associations between agents.

## Introduction

On a global level, milk is part of the diet of 6 billion people, of which the majority live in developing countries (FAO, 2019). The goat, a small ruminant, was the first farm animal to be domesticated (8000 BC, Ganj Darech), today known as Iran (Boyazoglu et al., 2005). Goat milk production has been growing steadily over the past 20 years due to the recognition of its nutritional values and nutraceutical properties (Kumar et al., 2016). Mastitis, or intramammary infection (IMI; Menzies and Ramanoon, 2001; Sar et al., 2018), is primarily caused by bacterial intramammary infection and is the most relevant small ruminant disease, causing severe economic losses to the dairy industry worldwide (Oikonomou et al., 2014; Dore et al., 2016) risk to public health due to the presence of pathogens and toxins released, as well as antimicrobial residues (Contreras et al., 2007).

Most cases of IMI are chronic, making them difficult to treat and prone to resurgence. Frequently, they are accompanied by long-lasting cost-intensive antibiotic treatment and premature culling (White and Hinckley, 1999; Menzies and Ramanoon, 2001; Schukken et al., 2011; Grunert et al., 2018); occasionally, the animals die if not properly medicated. In dairy animals, IMI can manifest itself in clinical forms of varying levels of severity, according with symptoms, otherwise, with a total absence of visible macroscopic signs of the disease, in the form of a subclinical infection (Côté-Gravel and Malouin, 2019). Several bacterial pathogens can cause IMI, but *Staphylococcus* spp. are the most frequently diagnosed causal microorganisms in goats and sheep (Contreras et al., 2007). Other pathogens, such as *Streptococcus* spp., the *Enterobacteriaceae* family, *Pseudomonas aeruginosa*, *Mannheimia haemolytica*, *Corynebacterium* spp., and fungi, can produce IMI in small ruminants, albeit with lower occurrence rates. The high diversity of microorganisms, mainly IMI-causing bacteria, makes treatment and control in human and veterinary medicine difficult (White and Hinckley, 1999; Contreras et al., 2007).

Microbiome studies of goats (McInnis et al., 2015; Zhang et al., 2017; Polveiro et al., 2020) and cows milk (Oikonomou et al., 2014; Addis et al., 2016; Bonsaglia et al., 2017) in several situations have demonstrated the complexity of the interactions of pathogens and commensals present in situations of health and illness. Diseases can be the consequence of a sum of many complex variables that interact, which can go beyond the mere interaction between host, pathogen and environment, as we know it (Hedrick, 1998), and milk omics analyzes have unravel new variables for understanding the different types of mastitis. Such novel research approach has allowed the reconstruction of systems approaching this infectious disease (Garira, 2019; Eckhardt et al., 2020). Few studies have revealed microbiota in goat milk; however, the main bacterial phyla found are *Proteobacteria*, *Actinobacteria*, *Firmicutes*, and *Bacteroidetes*, as well as a variety of genera such as *Acinetobacter*, *Agrobacterium*, *Alkalibacterium*, *Bacteroides*, *Bacillus*, *Enterobacter*, *Escherichia*/*Shigella*, *Fusobacterium*, *Klebsiella*, *Massilia*, *Micrococcus*, *Pseudomonas*, *Phyllobacterium*, *Rhodococcus*, *Staphylococcus*, *Stenotrophomonas*, *Stenotrophomonas*, *Shewanella*, *Streptococcus*, and *Yersinia* (McInnis et al., 2015; Zhang et al., 2017; Polveiro et al., 2020).

The objectives of this study were to generate knowledge of the microbiome of goat milk using samples from healthy goats and those diagnosed with subclinical, clinical, and gangrenous mastitis, characterized by 16S rRNA amplicon sequencing. First, we aimed to analyze and compare the populations of bacteria between healthy groups and types of mastitis; subsequently, we described the populations found among the groups and determined the most important agents among them. Finally, we performed predictive functional profiling of microbial communities and compared the metabolic and functional profiles of the bacteria.

## Materials and methods

### Criteria for the selection of animals and sampling

We collected 72 samples of milk from goats from six microregions and from 11 different goat herds from the Zona da Mata of the state of Minas Gerais, Brazil (Supplementary Table S1). All samples were collected in 2014 on commercial properties where the animals were raised in intensive milk production systems. Milk samples were segregated according to the clinical status of the animal, namely clinically healthy animals and animals with different types of mastitis. Thus, were used 12 samples of milk from animals diagnosed as clinically healthy (H0) and thus determined as control animals, 42 samples from animals with subclinical mastitis (M1), 16 samples from animals with clinical mastitis (M2), and 2 samples from animals diagnosed with gangrenous mastitis (M4; Supplementary Table S1). The management system of the herds used in this study was an intensive production system, in which the animals were kept stabled on slatted floor; most of the animals were of the Saanen goat breed. The animals had no clinical history with previous antibiotic therapy. Milk samples were collected by a trained veterinarian member of the research team, following the standard recommendations of the National Mastitis Council’s Laboratory Handbook on Bovine Mastitis (Hogan et al., 1999). Approximately 15 ml of milk was collected before milking and after the external cleaning of the ceiling with alcohol 70 (ethyl alcohol hydrate 70° INPM); the first jets of milk were discarded, and the teats of the animal were dried with paper towels. The samples were immediately refrigerated at 4–7°C, transported to the Laboratory of Bacterial Diseases (LDBAC) at the Veterinary Department of the UFV on ice, and 2-mL milk aliquots were stored at −80°C until further DNA extraction.

The pre-requisites for healthy control (H0) animals were that they did not present any signs of clinical mastitis during the physical examination, scored 0 and + 1 in the CMT (California Mastitis Test), and were negative in the bacterial culture, as detailed below.

The California Mastitis Test (CMT; Perrin et al., 1997), the evaluation of clinical breast signs, and bacteriological tests were used as parameters to diagnose IMI. The CMT reactions were performed with 2.5 ml of milk mixed with 2.5 ml of CMT reagent, and the CMT scores were graded (Perrin et al., 1997). The reaction was graded by the intensity of gel formation and color change. The milk samples with CMT scores of 0 and +1 were considered as negative, and those with a score of +2 or +3 were considered positive (Perrin et al., 1997). Animals were considered positive for M1 when negative in the evaluation of clinical signs and positive in bacteriological tests and with a score of +2 or +3 in the CMT.

The animals diagnosed with IMI, M2, and M4 were first evaluated for signs of clinical mastitis and the presence of at least visually abnormal milk (i.e., the presence of flakes, clots, blood, or serous milk). Changes in the mammary gland were also evaluated, such as an increased volume and body temperature as well as pain, redness during forestripping performed at the milking parlor, in addition to systemic clinical signs, in the presence of a veterinarian. The diagnosis of animals in M4 considered the previous conditions as well as the presentation of the udder in a bluish color and with an edematous aspect (Rainard et al., 2018). In addition, to fit into these M2 and M4 statuses, the animals were positive in bacteriological tests and scored +2 or +3 in the CMT.

### Bacteriological examination

For the isolation of the bacteria, 100-μL samples of pre-homogenized milk were used for full aerobic bacteriological culture and spread on Columbia agar supplemented with 5% sheep blood. All milk samples were directly cultured for aerobic bacteria using described standard culture techniques (Aarestrup et al., 1995; Quinn et al., 2003; Versalovic et al., 2011). Plates were read after 24, 48, and 72 h. Plates with more than three colonies after 48–72 h of incubation at 37°C were considered positive (M1, M2, and M4), according to the protocols mentioned above, from an individual milk sample; for H0 samples, plates that did not demonstrate bacterial development were considered negative (Buelow et al., 1996; Quinn et al., 2011).

### DNA extraction, amplification of the 16S rRNA gene, and sequencing

The total DNA of the milk samples was extracted using the QIAmp DNA kit min (QIAGEN, Valencia, CA), following the process protocol ‘Blood or Body Fluid Spin Protocol’ (Spin Protocol), with modifications described by Kuehn et al. (2013). The concentration and purity of the DNA were quantified by spectroscopy (optical density) on a NanoDrop® Thermo Fisher Scientific Spectrophotometer (Waltham, Massachusetts, United States; Oikonomou et al., 2012). Samples of extracted DNA were sent to the Argonne National Laboratory (Lemont, IL, United States) in an ice-dry isothermal box at −78°C for sequencing.

In the Argonne Laboratory (Argonne, IL, United States), the V4 hypervariable region of the bacterial 16S rRNA gene was amplified from genomic DNA by polymerase chain reaction using the primers 515F and 806R, optimized for the Illumina MiSeq platform (Illumina Inc., San Diego, CA; Caporaso et al., 2012), with MiSeq Reagent Kit V2.

### Sequence and bioinformatics analyses

The sequences were demultiplexed using the ‘Idemp’ program.^1^ The package ‘DADA2’ pipeline (version 1.8) in R (R Team Core, 2018) was used to infer the amplicon sequence variant (ASV) present in each sample (Callahan et al., 2016). The ASV methods have demonstrated sensitivity and specificity as good or better than those of operational taxonomic units (OTUs), identifying the distinction of sequence variants by as little as one nucleotide(Callahan et al., 2017). Bioinformatics processing largely followed the DADA2 tutorial.^2^ Forward and reverse read pairs were trimmed and filtered, truncated at 150 nt, and reverse read at 150 nt, with up to two bases of ambiguous errors allowed; each read was required to have less than two expected errors based on their quality scores. The ASVs were independently inferred from the forward and reverse reads of each sample, using the run-specific error rates, and read pairs were merged. Chimeras were identified for each sample and removed if identified in a sufficient fraction of the samples by the method consensus. Taxonomic assignment was performed against the Silva v. 132 database, using the implementation of the Ribosomal Database Project (RDP) Classifier, a naïve Bayesian classifier, available in the package ‘DADA2’ in R in default parameters (Wang et al., 2007; Quast et al., 2013).

All statistical analyses were carried out by using several packages and functions implemented in R 4.0.3 (R Team Core, 2018). We did not analyze non-rarefied data due to the characteristics of our analyses in the data (McMurdie and Holmes, 2014; Weiss et al., 2017; Zaheer et al., 2018). Using the package ‘*phyloseq’* (McMurdie and Holmes, 2013), however, we polished the data with the removal of any ASVs without a bacterial phylum assignment, assigned as *Archaea*, chloroplast, or mitochondrial origin. To simplify downstream analyses and to reduce the noise of the analyses, we applied a prevalence and abundance threshold for bacterial ASVs, in which taxa were kept only if they were found at a minimum frequency of 100 in at least one sample.

### Diversity analysis and microbiota composition

Alpha diversity was analyzed in the package *phyloseq* (McMurdie and Holmes, 2013), using metrics of the indices Shannon diversity (Shannon, 1948), Chao1 richness (Chao, 1984), and Observed Species in the R statistical software (R Team Core, 2018). To test for normality statistically, we ran the Shapiro–Wilk test of normality before comparing different mastitis alpha-diversity values. The values of the indices for different types of mastitis (taken from all groups, H0, M1, M2, and M4), were compared by ANOVA (α < 0.05), followed by the Tukey *post hoc* test using the package *vegan* (Oksanen et al., 2019).

The dissimilarity in community structure between different mastitis types was assessed by principal coordinate ordination using Bray–Curtis, unweighted and weighted UniFrac metrics, by performing non-metric multidimensional scaling (NMDS; Bray and Curtis, 1957) and with canonical analysis of principal coordinates (CAP; Anderson and Willis, 2003), followed by the analysis of differences for ANOVA. Deeper analyses with permutational multivariate analysis of variance (PERMANOVA) were performed for differences in the communities among H0, M1, M2, and M4, which were conducted using the function adonis from the package *vegan* (Oksanen et al., 2019) and Bray–Curtis dissimilarity over 1,000 permutations. Pairwise *post hoc* tests were conducted using the function pairwise.adonis from the package *pairwiseadonis* (Martinez Arbizu, 2020) with Bonferroni correction to calculate the statistical significance.

The microbiota composition in the bar graph was analyzed using the Phyloseq package (McMurdie and Holmes, 2013), ‘Microbial’^3^ and ggplot2 (Wickham, 2016) in R. The R package *metacoder* (Foster et al., 2017) was used for representing the taxonomic abundance as a differential heat tree, along with cladograms of the taxonomy, using a Wilcox rank-sum test followed by a Benjamini-Hochberg (FDR) correction for multiple comparisons. The packages *MicrobiotaProcess* (Xu and Yu, 2020) and *VennDiagram* (Chen and Boutros, 2011) were used to build a Venn diagram with the different types of mastitis.

### Predictive functional profiling of microbial communities

The *PICRUSt2* software (Douglas et al., 2020) was used to infer functional profiling of microbial communities by means of a set of minimum pathways identified by the *MinPath* (Minimal set of Pathways) tool (Ye and Doak, 2009) and pathway definitions by gene family provided by the *MetaCyc* metabolic database (Caspi et al., 2018). The *STAMP* (Parks et al., 2014) software package was used to analyze the metabolic potential of the microbial communities. The groups (H0, M1, M2, M4) were compared using an ANOVA, followed by a Tukey–Kramer post-hoc test (0.95), with statistical significance accepted when the *p*-value ≤0.01 and Benjamin–Hochberg FDR for correction.

## Results

### Summary of treatments, microbiological tests, and sequencing

We used 72 samples of goat milk, 12 samples from healthy controls (H0), 42 samples from animals diagnosed with subclinical mastitis (M1), 16 samples from animals diagnosed with clinical mastitis (M2), and 2 samples from animals diagnosed with gangrenous mastitis (M4). Of these, 60 samples, M1, M2, and M4, contained multiple microorganisms, whereas 12 (H0) samples did not show bacteriological growth in the culture medium (Supplementary Table S1).

Goat milk samples were collected and sequenced in the V4 region of the 16S rRNA gene; quality-filtered reads were demultiplexed, and a total of 2,298,725 sequences were used for downstream analyses (mean = 31,926,736 ± SD = 11,357,791 reads/sample). The median length for all reads was 254 bp. Overall, 869 taxa identified were used in the analyses.

### Alpha diversity of milk microbiota of goats with mastitis and of healthy goats

**Figure 1:**
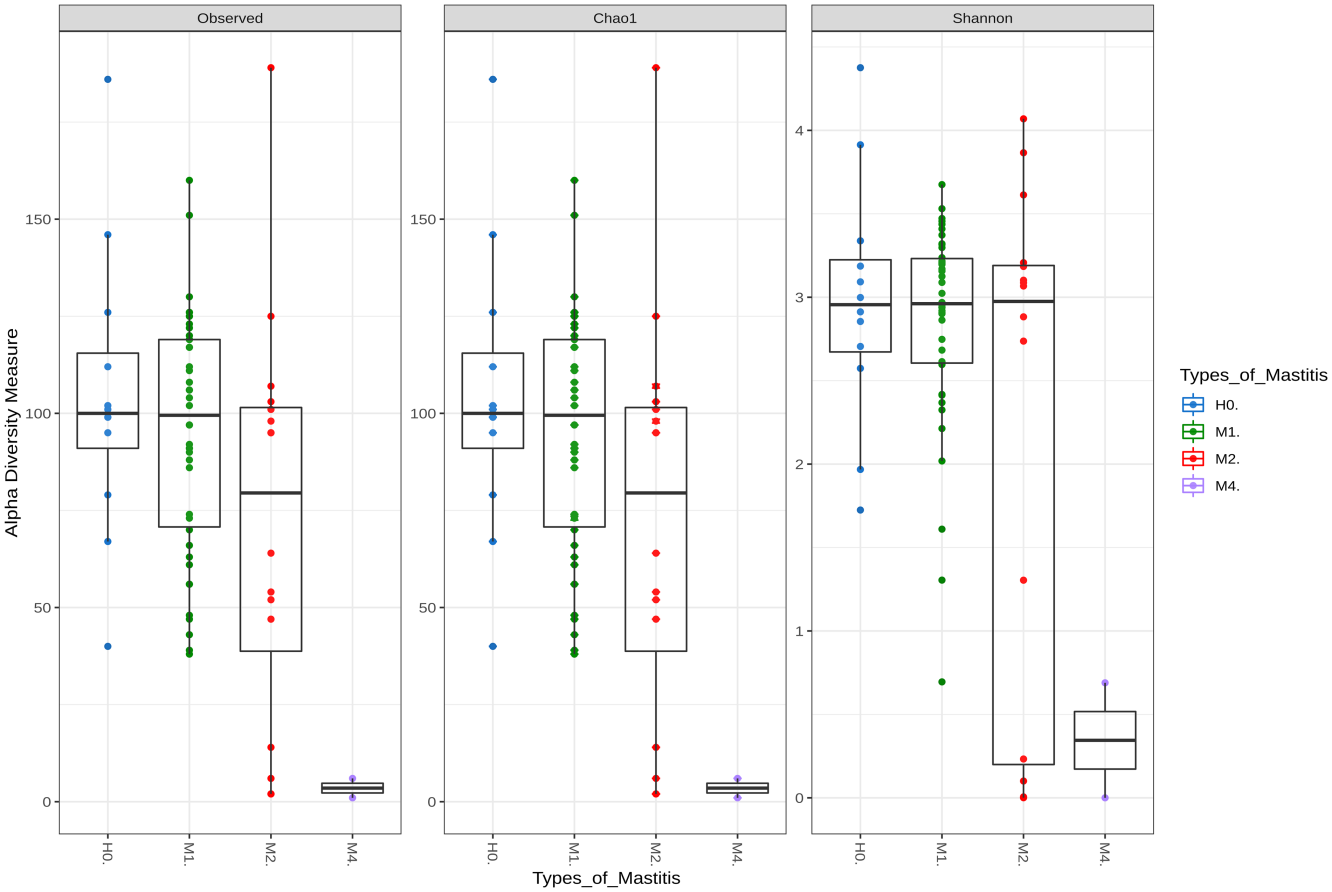
Alpha diversity indices for healthy groups (H0), subclinical mastitis (M1), clinical mastitis (M2), and gangrenous mastitis (M4). Each color represents a group of mastitis type by disease severity, with the Chao1 index representing the richness index and Shannon the sample diversity index. Statistical differences were significant (*p*-value <0.01) between M4–M1 and M4–H0.

Richness and diversity were analyzed to assess whether any divergence was observed across groups. The Chao 1 and Shannon measures, which are related to richness and diversity, respectively, were statistically different among treatment groups: M4–M1 (*p* < 0.01) and M4–H0 (*p* < 0.01; Supplementary Tables S2–S4; Figure 1).

The richness and diversity of M1 differed slightly in its extremes from those of the control group H0, which denotes a slight dysbiosis of the mammary gland, presented with this type of mastitis. On the other hand, M2 showed a high reduction in bacterial microbiota richness, even though it was not statistically significant, in relation to the healthy animals of H0. The animals diagnosed with gangrenous in M4 were the ones that differed graphically and numerically in richness and diversity from those with other types of mastitis, which corroborates the clinical symptoms in these animals, with tissue loss and the ability to produce milk in the udder, in addition to the almost complete destruction of the microbiota.

### Differences in microbial composition among groups based on beta diversity

**Figure 2:**
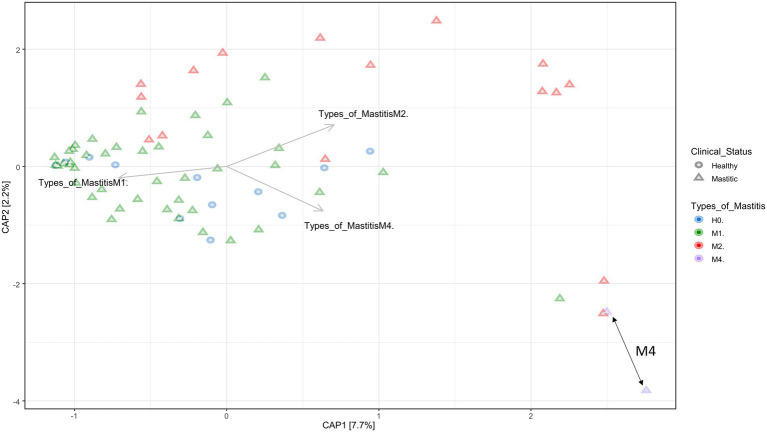
Canonical Principal Coordinate Analysis (CAP) built on a Bray–Curtis dissimilarity matrix with groups of healthy animals (H0), animals with subclinical mastitis (M1), animals with clinical mastitis (M2), and animals with gangrenous mastitis (M4). The forms depict healthy animals and animals with mastitis, and the colors indicate the mastitis type. The order of the arrows demonstrates the formation of groups of individuals selected in different coordinates, denoting the dissimilarity and similarity of microbiota composition among samples and groups, according to the type of mastitis. The double arrows on the lower right demonstrate the most isolated grouping of animals belonging to the M4 group. The *p*-value = 0.001 was obtained by means of ANOVA.

The dissimilarities between the groups of the different types of mastitis can be seen in Figure 2, showing the canonical analysis of principal coordinates (CAP). Although there is no clear separation in Figure 2, there was a significant difference among the groups, with a *p*-value = 0.001 (Supplementary Table S5). On the other hand, as seen in the Supplementary Figures S1, S2, that consider phylogenetic distance measurements, the groups were separated based on UniFrac unweighted distances, considering only presence and absence of species information, and UniFrac weighted distances, which use species abundance information.

Although the group separations were not clear, dysbiosis apparently caused an effect of distance from a central point where the samples of healthy animals were distributed, going in a centrifugal direction to the graph, especially when we evaluated the presence and absence of species (Supplementary Figure S2). This demonstrates a possible distance between the species of the microbiota as the degree of severity of mastitis increases, that is, the dysbiosis is accentuated. Other statistical analyses presented in Supplementary Table S5 show significant differences for the occurrence of separations between groups, H0 vs. M4, M1 vs. M2, and between M1 vs. M4. In addition, the statistical correction of Bonferroni values demonstrated an evident dissimilarity between M1 and M2, referring to subclinical and clinical mastitis, respectively.

### Differences in the composition of the bacterial microbiota for each group by mastitis type

**Figure 3:**
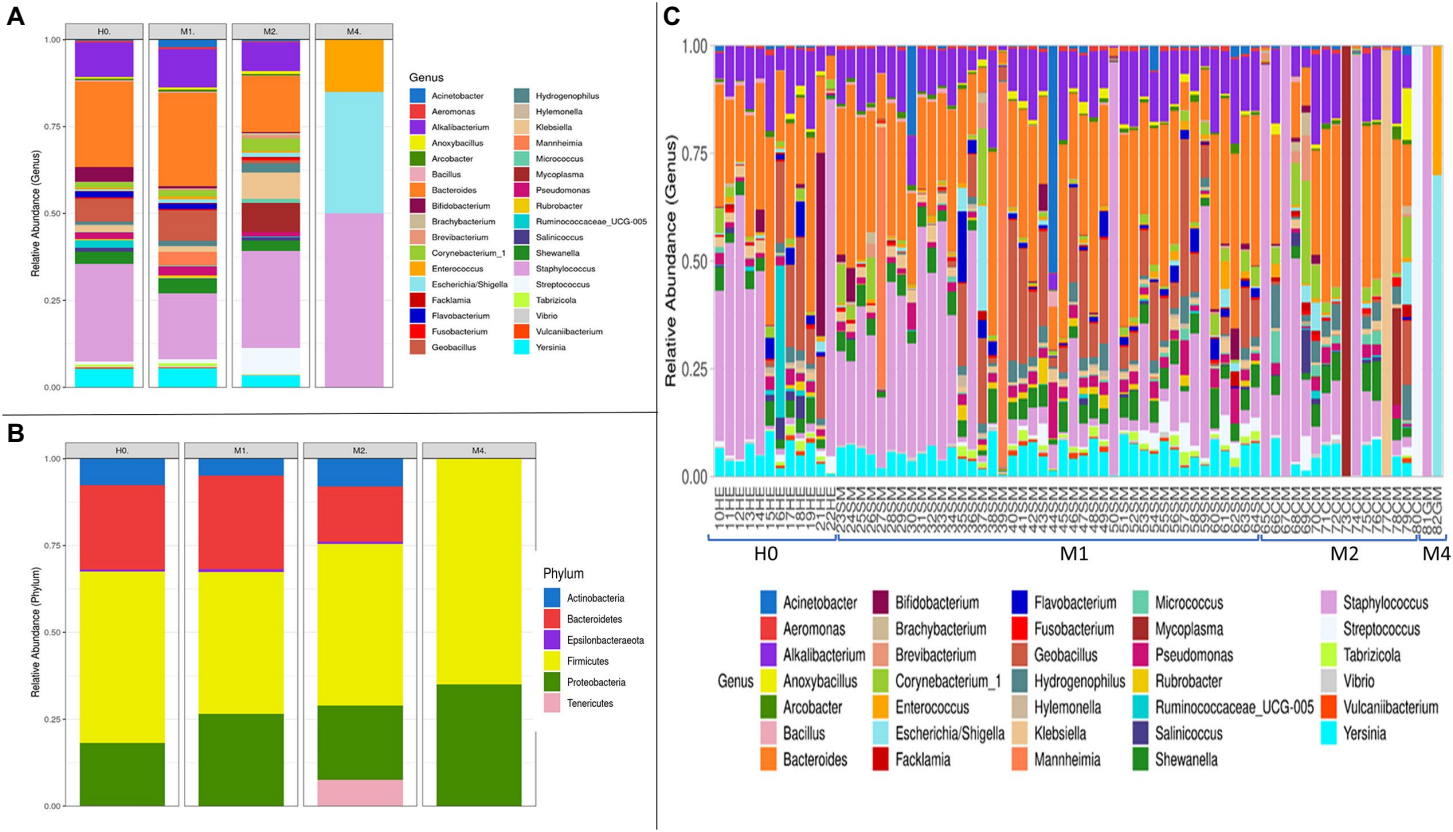
Bacterial microbiota composition in terms of relative abundance at phylum and genus levels, in the groups of goat milk samples separated into clinically healthy (H0), subclinical mastitis (M1), clinical mastitis (M2), and gangrenous mastitis (M4): **(A)** Taxonomic composition of the 35 main bacterial genera and taxa with different abundances among the different types of mastitis, with each color corresponding to a different genus. **(B)** Taxonomic composition of the six main phyla and differentially abundant bacterial taxa, with each color corresponding to a phylum, in the different types of mastitis and healthy control animals. **(C)** Taxonomic composition of the 45 main genera and differentially abundant bacterial taxa, with each color corresponding to a different genus and subdivided by independent samples, which relate to clinically healthy (HE) animals and animals diagnosed with subclinical mastitis (SM), clinical mastitis (CM), and gangrenous mastitis (GM).

The graphs in Figure 3 refer to the microbial compositions found in animals with different types of mastitis and in clinically healthy animals. Among the genera that stand out in Figure 3A, we found the highest abundance among the *Staphylococcus* sp. groups, with variable rates of 28.41, 21.05, 29.82, and 46.60% for groups H0, M1, M2, and M4, respectively. Interestingly, *Bifidobacterium* sp. was the fifth most abundant genus in H0, and there was a gradual reduction of 4.25, 0.49, 0.18 to 0.00% in the milk of animals from groups H0, M1, M2, and M4, respectively. Another relevant finding was the presence of 17.98% for the genus *Mycoplasma* sp. in group M2, in samples 70CM, 73CM, 76CM, and 78CM (Figure 3C), which demonstrates that this genus may have helped to compromise the udder microbiota of these animals. In Figure 3A, for the H0 group, the most abundant genera after *Staphylococcus* sp. were *Bacteroides* sp. (25.00%), *Alkalibacterium* sp. (9.86%), *Geobacillus* sp. (6.11%), *Yersinia* sp. (5.47%), *Bifidobacterium* sp. (4.25%), *Shewanella* sp. (3.57%), and *Pseudomonas* sp. (1.92%). The other percentages referring to the remaining groups are provided in Supplementary sheet 1.

Among the six main phyla that we found among the groups described in Figure 3B, we observed large oscillations between H0 and M1 for the phylum *Proteobacteria*, whose percentages ranged from 18.19 to 26.15%, respectively. The phylum *Bacteroidetes* was reduced from 25% in H0 and 26% in M1 to 12% in M2; it was not detected in M4. On the other hand, the phylum *Tenericutes* increased from 0.05% in H0 and 0.04% in M1 to 16.09% in M2, most likely because of the increased detection of the genus *Mycoplasma* sp. in M2. Interestingly, the samples for the type of gangrenous mastitis (M4) in Figure 3C showed a high abundance of reads of up to two genus, *Staphylococcus* sp. (81GM) and in another, of the genera (82GM) *Escherichia* sp./*Shigella* sp. and *Enterococcus* sp. The classification technique could not distinguish the genera *Escherichia* sp./*Shigella* sp. in this study. The microbiota of the M4 group was reduced to a few abundant pathogenic organisms, completely mischaracterizing the microbiota in relation to samples from animals with other types of mastitis (M1, M2) and from clinically healthy animals (H0). Therefore, in line with Figure 1, the gangrenous mastitis shown in Figure 3 almost completely removed the diversity and richness of the udder microbiota.

**Figure 4:**
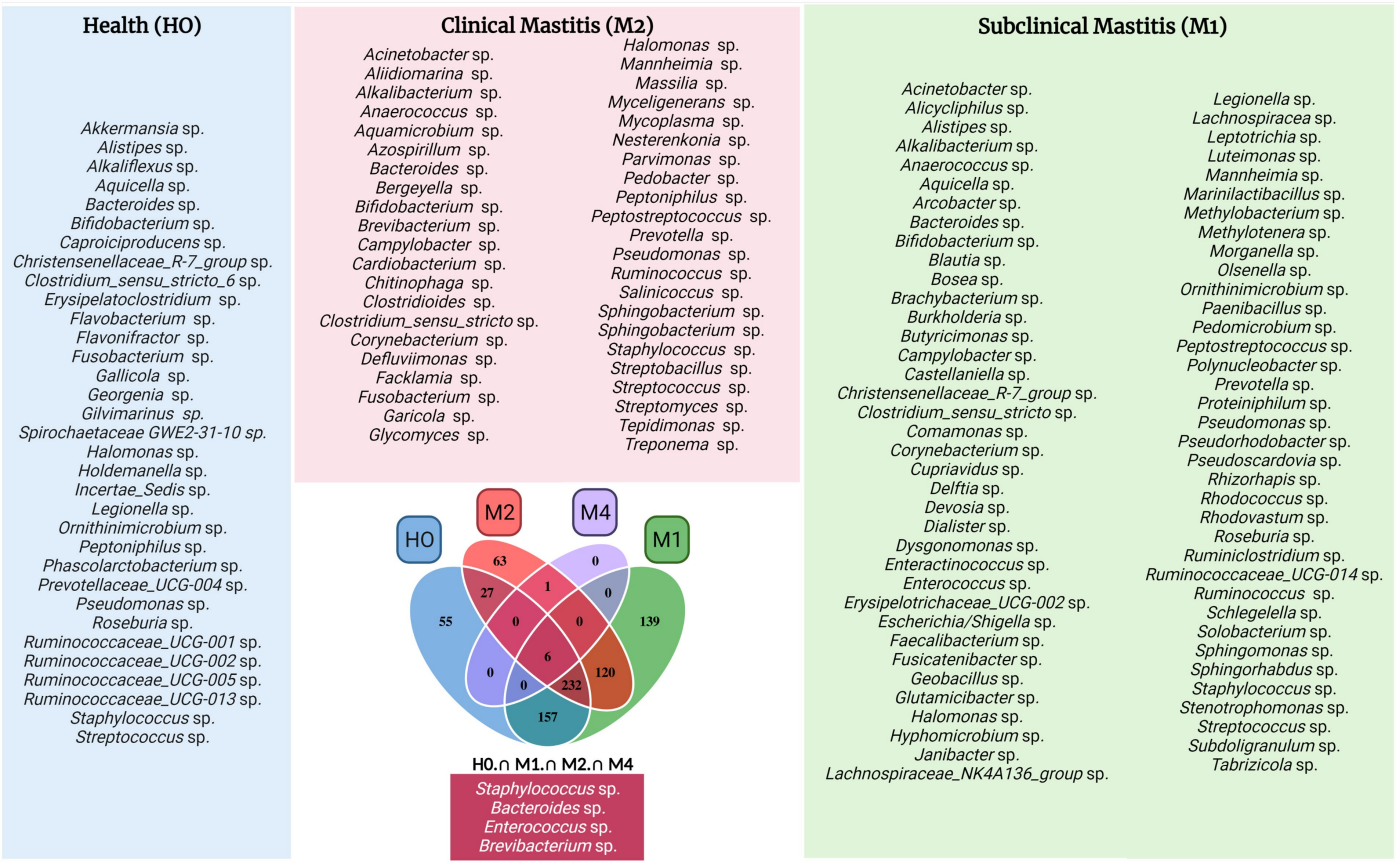
Venn chart with taxonomic groups of bacteria divided by mastitis types and clinically healthy animals, with each table distributed to each genus and other taxonomic ranks. The groups are equally distributed in different colors, as well as their intercalations, with H0 corresponding to healthy controls, M1 to animals diagnosed with subclinical mastitis, M2 to animals with clinical mastitis, and M4 to animals diagnosed with gangrenous mastitis. The frames on the sides of the Venn diagram are labelled with the colors of their respective groupings of the central Venn figure and identify each taxonomic rank that has been cataloged for their groups. Some genera or ranks may have the same name, but they are different amplicon sequence variants (ASVs), that is, different species and subspecies are possible. The chart located below the Veen graph is composed of taxa common to all groups, with *Staphylococcus* sp. and *Enterococcus* sp. having one more ASV each, resulting in a total of six taxa. The graph was created with BioRender.com.

In a more robust analysis, Figure 4 demonstrates the variation of microbiome taxa among animals with different types of mastitis and healthy animals, indicating significant differences among the median proportion of reads for each group of samples determined. Among these, *Prevotella* spp. (H0-M1), Ruminococcaceae (H0-M1), *Prevotella ruminicola* sp. (H0-M1), *Providencia* sp. (M1-M4), *Nesterenkonia* sp. (H0-M1), *Rubrobacter* sp. (M1-M2), *Flavobacterium* sp. (M1-M2), *Stearothermophilus* sp. (M1-M2), *Tabrizicola* sp. (M1-M2), *Acetobacter* sp. (M1-M2), *Vulcaniibacterium* sp. (M1-M2), *Geobacillus* sp. (M1-M2), *Polynucleobacter* sp. (M1-M2), and *Yersinia intermedia* spp. (M1-M2) presented the highest significance among the 365 genera identified (*p*-value 0.05). After the Benjamini-Hochberg correction (FDR) for multiple tests, we identified the first four taxa mentioned above as the most important among the groups. The other significant taxa are listed in Supplementary sheet 2.

Among the genera mentioned above in Figure 3, with high relative percentage fluctuations, some were significant (Figure 4) in one or more groups, such as *Alkalibacterium* sp. (M1–M4, H0–M4, *p*-value 0.05), *Mycoplasma* sp. (M1–M2, *p*-value 0.05), *Enterococcus* sp. (M1–M2, *p*-value 0.05), as well as *Bacteroides* sp. and *Pseudomonas* sp. (M1–M2, M1–M4, H0–M4, *p*-value 0.05).

### Distinction of microbiota by the Venn diagram

**Figure 5:**
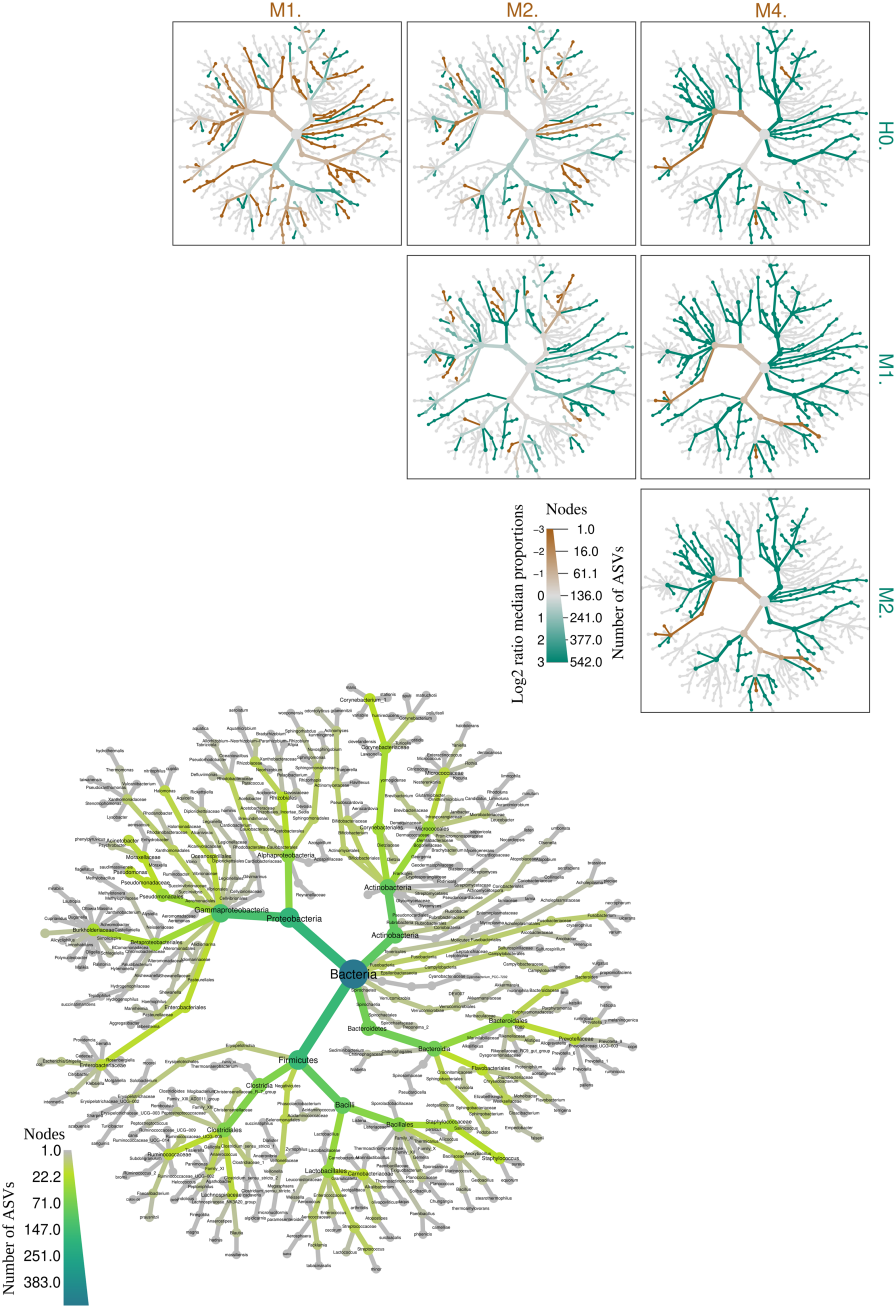
Heat tree illustrating the general taxonomy of the milk bacterial community in all types of mastitis and healthy control animals. The larger heat tree, lower left, shows the names of the taxa found. The size and color of nodes and edges are correlated with the abundances or numbers of amplicon sequence variants (ASVs) of organisms in the communities found. The smaller heat trees, on the right and top, illustrate comparisons between groups of different types of mastitis and healthy control animals. The color intensity is related to the log-2 ratio of the difference in median proportions and to the Wilcox test applied to the readings among each group: healthy (H0), subclinical mastitis (M1), clinical mastitis (M2), and gangrenous mastitis (M4). The brown taxa indicate an enrichment in the different types of mastitis; healthy animals listed at the top of the graph, and green refers to the opposite in the other comparative group. In gray, the nodes are equally present in both compartments.

The distribution and exclusivity of genera and other taxonomic ranks among groups can be seen in the Venn diagram (Figure 5). All taxa of bacteria belonging to each treatment group are listed, as well as their intercalations among groups. The smallest number of bacteria belonging to a single group is in the H0 group was 55, followed by 63 in M2 and 139 in M1. Group M4, belonging to the gangrenous mastitis group, did not have a single exclusive taxon. This may be because this more serious condition of mastitis is a development of the evolution or advanced stage of the disease, of the clinical conditions that may precede it, such as subclinical and clinical mastitis, and therefore, the bacteria of these other clinical conditions become dominant in the microbiota of animals diagnosed with mastitis type M4.

While H0 had 157 bacterial taxa in common with M1, it only had 27 in common with M2, which may be a sign of increased dysbiosis in the mammary gland. This indicates that the increase in the intense inflammatory activity of mastitis in goats, when trying to control the development of new taxa in the mammary gland, in the development of M1, leads to a reduction in the richness of M2, as shown in Figure 1, breaking with equilibrium in H0 and, consequently, culminating in an abrupt reduction of microorganisms in M4. This supports the findings shown in Figure 4.

Group M4 group only had one exclusive taxon in common with group M2 group. This demonstrates that group M4 represents a picture of the clinical evolution of the previous ones and of rupture of the microbiota of the mammary gland in relation to clinically healthy animals in H0. The genus *Staphylococcus* sp. appeared more frequently among the groups; however, the species of this genus can perform different functions in microbiota. Some of the genera shown appeared repeatedly in the same groups and in others due to the genus classification through the pipeline package ‘DADA2’, which performs the distinction of sequence variants by only one nucleotide, with a better distinction of genus and species.

### Predicted functional metagenome in personality groups

**Figure 6:**
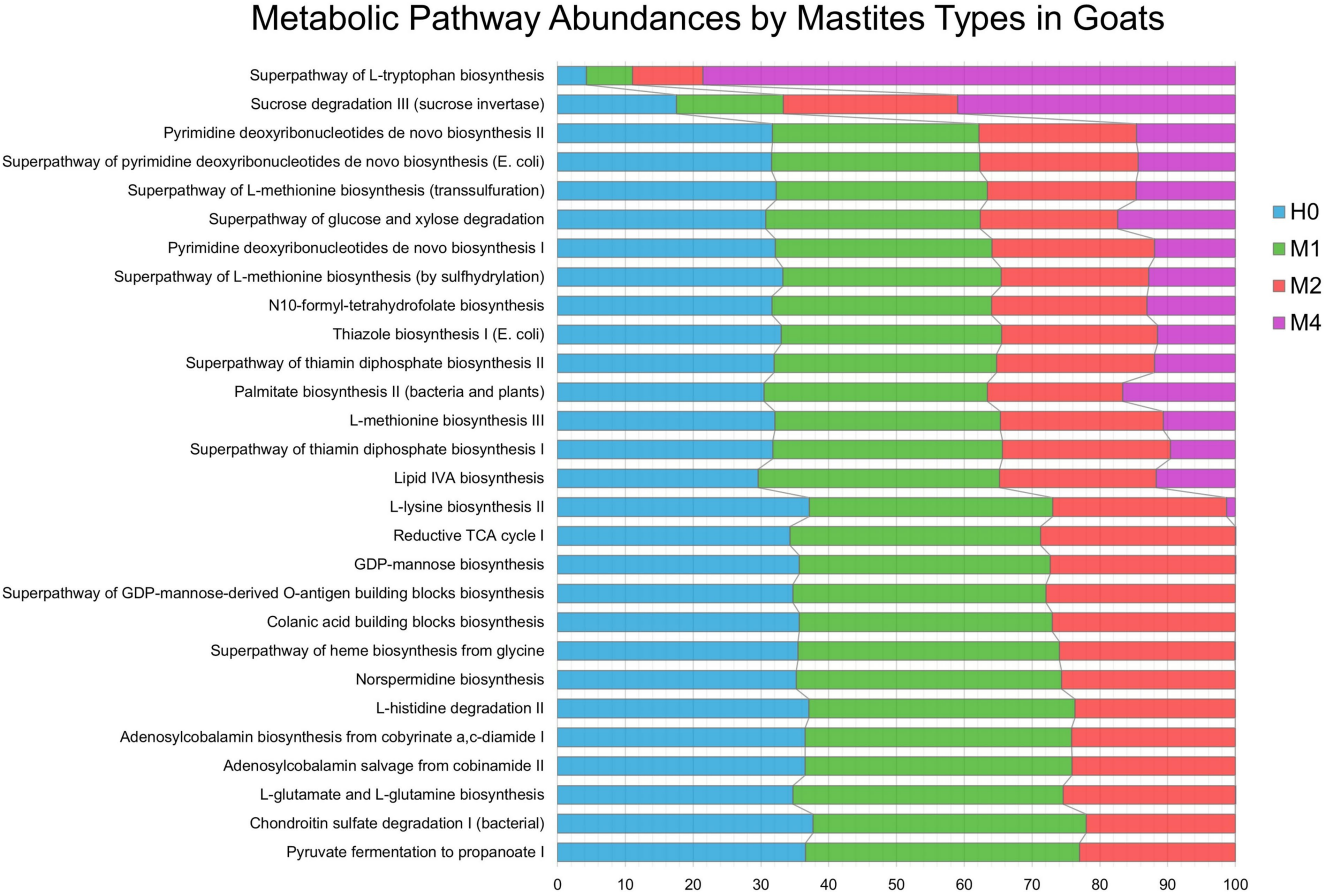
Metabolic Pathway Abundances by Mastitis Types in Goats. Characteristics of microbial functional pathways in different samples of goat’s milk from groups of healthy animals and animals with different types of mastitis. Prediction of goat milk microbiota function and metabolic pathways from healthy controls (H0 – blue), animals with subclinical mastitis (M1 – green), animals with clinical mastitis (M2 – red), and animals with gangrenous mastitis (M4 – purple) by pathway definitions *via* the gene family, provided by the MetaCyc metabolic database, transformed into relative frequencies and percentages. Comparison of predicted metabolite pathways was performed using the PICRUST and STAMP programs. Significant pathways were selected using ANOVA (*p*-value <0.01), with Tukey–Kramer test (0.95) and Benjamin–Hochberg FDR for correction.

Figure 6, we show the significant changes of the predicted metabolic pathways for the microbiota in each sample belonging to the groups of controls (H0), subclinical mastitis (M1), clinical mastitis (M2), and gangrenous mastitis (M4), according to the MetaCyc metabolic database. Among the groups, 28 important metabolite pathways were identified (Figure 6), of which we highlight the superpathway of L-tryptophan biosynthesis, which showed a continuous growth in absolute numbers as the severity of mastitis increased from H0 to M1, M2, and M4. The sucrose degradation III (sucrose invertase) pathway slightly decreased from H0 to M1 but showed a significant increase from M1 to M2 and M4. The greatest variations from H0 to M1 occurred in the lipid IVA biosynthesis and L-glutamate and L-glutamine biosynthesis pathways; from M1 to M2, there were significant variations in pyruvate fermentation to propanoate I and chondroitin sulfate degradation I (bacterial). The other pathways showed a slight increase or remained stable from H0 to M1, decreased to M2, and then decreased abruptly to M4.

## Discussion

Milk constitutes a complex microbiota, which can be widely altered by different factors inherent to the species, breed, and health of the animal, as well as by different types of mastitis (Bhatt et al., 2012; Oikonomou et al., 2014; Li et al., 2018; Hoque et al., 2019; Polveiro et al., 2020). Microorganisms present in milk greatly influence the safety and quality of dairy products (Li et al., 2018), and investigating the composition and structure of groups of bacteria that can be formed in different types of mastitis can reveal patterns of population signatures that help to better understand the development and outcome of infections of the mammary gland. Our data suggest that the progression of subclinical mastitis, from clinical to gangrenous, plays crucial roles in the composition and structure of the bacterial microbiota of goat milk, modifying the richness and diversity and gradually accentuating the condition of dysbiosis, in addition to causing changes in relation to the indigenous microbiota of clinically healthy animals.

Studies with cattle (Oikonomou et al., 2014; Sokolov et al., 2021), using the V1–V2 and V3–V4 regions, and buffaloes (Catozzi et al., 2017), with respective sequencing of the V1–V2 region, have already highlighted differences among groups of mastitis in alpha diversity, although they were not statistically significant (Oikonomou et al., 2014), as observed in this study with the amplification of the V4 region. The significant distortions of diversity and richness caused in the microbiota by gangrenous mastitis demonstrates that there was an almost complete elimination of bacteria from the microbiota of these affected animals, resulting in a high abundance of pathogenic genera. Generally, this type of mastitis is associated with the agents *Staphylococcus* sp. (Peer and Bhattacharyya, 2007; Sabuncu et al., 2015), *Escherichia coli* (Ameh et al., 1994), *Bacillus* sp. (Mavangira et al., 2013) or by co-infection of bacteria (Ribeiro et al., 2007). Gangrenous mastitis results from an exacerbated inflammatory and infectious process in the mammary glands, which may not be directly associated with the recruitment of leukocytes but with the late response time and the production of exotoxins. (Rainard et al., 2018). In addition, it is a serious clinical condition, leading to the loss of milk production capacity, mastectomy, culling, and high lethality (Abu-Samra et al., 1988; Peer and Bhattacharyya, 2007; Ribeiro et al., 2007; Sabuncu et al., 2015; Rainard et al., 2018). In this study, we detected an association between *Escherichia* sp./*Shigella* sp. and *Enterococcus* sp., in addition to an infection with a high abundance of *Staphylococcus* sp.

The distribution of the types of mastitis in the beta diversity analyses shows the separation of the total and partial groups; however, this difficulty in clearly separating the groups and has already been demonstrated in other studies with cattle (Porcellato et al., 2020; Sokolov et al., 2021), and in this study, it may have occurred because those certain samples were collected in the transition of dysbiosis states between types of mastitis and healthy controls. This phenomenon can interfere with several dynamics (Xia et al., 2018), increasing the severity from subclinical to clinical and gangrenous mastitis or jumping between the types, depending on several other factors inherent to the agents, such as location, host, environment, management, and the microbiota and its resilience to previous dysbiosis cases (Polveiro et al., 2020).

Based on our results, the genus *Staphylococcus* sp. plays an important role, with a high abundance, in the goat mammary gland microbiota in the three types of mastitis studied here, as well as in the microbiota of healthy animals. Among this genus, *Staphylococcus* Coagulase-Negative (SCN) and *Staphylococcus aureus* are the most frequently diagnosed causes of subclinical and clinical IMI in goats, respectively (Contreras et al., 2007; Dore et al., 2016). Previous studies have also found this genus in the milk microbiota of humans (Hunt et al., 2011; Fernández et al., 2020; Gryaznova et al., 2021), cows (Oikonomou et al., 2012, 2014) and healthy goats (Deinhofer and Pernthaner, 1995; Zhang et al., 2017; Polveiro et al., 2020). On the other hand, even though this agent is present in animals with mastitis or healthy animals, the species of *Staphylococcus* sp. that could not be taxonomically discriminated in this study, can be the key to characterizing its real role in dysbiosis in mastitis cases.

In this study, the milk of goats was mainly colonized by the phyla Firmicutes, Bacteroidetes, Actinobacteria, and Proteobacteria; they have also been found in the milk of other goats (Zhang et al., 2017), cows (Gryaznova et al., 2021), humans, and in human intestines (Urbaniak et al., 2016; Rinninella et al., 2019). Changes in the phylum Proteobacteria, as demonstrated here in the milk of healthy animals, in relation to subclinical mastitis, in the human microbiota are related to some population signatures in the microbiota in diseases (Rizzatti et al., 2017). The abundance of Bacteroidetes was greatly reduced in clinical mastitis and was not found in mastitis, which is in agreement with a previous study on cows (Gryaznova et al., 2021). This phylum contains commensals, mutualists, beneficial organisms, and species assisting the host in providing numerous health benefits (Zafar and Saier, 2021).

According to previous reports, there are about 80 different *Bifidobacterium* species (Parte, 2018; Wong et al., 2020). The genus *Bifidobacterium* sp., highly important in the microbiota of goat milk (Zhang et al., 2017), showed a decrease in abundance as mastitis severity increased. It has already been detected in the microbiota of raw milk (Quigley et al., 2013) and is among the most dominant taxa in studies on the healthy microbiota of human and bovine milk (Oikonomou et al., 2020). In addition, it is often detected in fermented dairy products (Milani et al., 2019), and isolated from various environments, such as human milk (Wong et al., 2020), human gut (Duranti et al., 2016), and bovine rumen (Biavati and Mattarelli, 1991). This has raised the hypothesis that the ability of bifidobacteria to adapt to specific environments is species-dependent (Alessandri et al., 2019; Duranti et al., 2020).

The genus *Prevotella* sp. and the family Ruminococcaceae, which differed between groups of healthy animals and animals with mastitis, generally stand out in the central microbiome of the cattle rumen (Henderson et al., 2015; Zhong et al., 2020), in the microbiota of cow’s milk associated with mastitis (Lima et al., 2017; Taponen et al., 2019; Gryaznova et al., 2021), and in the microbiota of milk from women associated with mastitis (Boix-Amorós et al., 2020). Hypothetically, these bacteria reach the milk microbiota in the mammary gland through an endogenous origin, which has been corroborated by different studies carried out in mice and cows (Addis et al., 2016; Ma et al., 2018; Hu et al., 2022). On the other hand, the genus *Providencia* sp., showed prominence among groups of subclinical and gangrenous mastitis; it has been detected in cow rumens (Stergiadis et al., 2021) and in the microbiota of milk in cows with mastitis (Saidi et al., 2013), as well as in sheep milk (Tvarožková et al., 2021) and goats milk from animals with mastitis (Mahlangu et al., 2018).

The genus *Alkalibacterium* sp., proposed by Ntougias and Russell (2001), stood out among the gangrenous, healthy, and subclinical mastitis groups. Members of this genus are typical lactic acid bacteria and can develop at different pH values; they have been isolated from fermented foods and beverages (Tamang et al., 2016), cheeses (Ishikawa et al., 2007, 2013; Ryssel et al., 2015; Yunita and Dodd, 2018), biofilms in olive samples (Benítez-Cabello et al., 2020), and marine environments (Ishikawa et al., 2009). The identification or isolation of the genus *Alkalibacterium* in any milk sample, especially from goats, although not yet widely reported in the literature, it was previously reported in another study by our group (Polveiro et al., 2020).

The sudden increase in the phylum *Tenericutes* in milk from goats with clinical-type mastitis was due to the genus *Mycoplasma* sp. and may have occurred due to infections or mycoplasmosis, which are highly prevalent in some areas and cause financial losses due to mortality or the need to cull animals, as well as a reduction in milk quality (Contreras et al., 2003).

We observed that the milk of healthy animals had a more specific microbiota, as discussed previously (Polveiro et al., 2020), with fewer exclusive microorganisms compared to milk from animals with subclinical and clinical mastitis. Subclinical mastitis develops with the advent of the introduction of new microorganisms in the microbiota of these animals. Consequently, with the evolution of the condition to clinical mastitis, depending on the immune response of the animals, other variables, and the various connections of these microorganisms through quorum-sensing systems (Wu and Luo, 2021), the selection of suitable microorganisms can be promoted, reducing the number of different taxa. Therefore, in subclinical mastitis, there is first an apparent increase in the richness of unique microorganisms, followed by a decrease in clinical mastitis. This microbial imbalance or difference in the microbial composition is treated as dysbiosis (Derakhshani et al., 2018).

The increase in L-tryptophan biosynthesis by the milk microbiota, described in the aggravation of mastitis in goats, is important. The metabolic pathways associated with tryptophan (Trp), as highlighted in this study, are important in the host–microbe interaction: on the one hand, they guarantee a positive symbiosis between the host and the Trp-synthesizing microbes; on the other hand, they can be used to deprive the host or, vice versa, Trp-auxotrophic pathogens of Trp, resulting in increased or decreased virulence, respectively (Costantini et al., 2020). The sucrose degradation III pathway (sucrose invertase) can be directly linked to the commensal or indigenous microbiota, being used to inhibit the growth of pathogenic bacteria by competition, as reported in studies with *Clostridioides* sp. (Fishbein et al., 2022). In this way, this pathway is always activated in the healthy microbiota and decreased as the mastitis worsens. The functional profiles of metabolites of the microbial communities are directly related to the loss of commensal or indigenous microbiota and the increase in the abundances of pathogenic microorganisms.

The microbial profiles in these samples reflect real-world clinical practices in the field with goats and therefore more accurately capture the bacterial composition of milk from animals with clinical diagnosis in different types of mastitis. However, our study has some limitations. In view of this, it is important to note that these analyses are limited to relative abundances, rather than absolute measures, of bacteria in goat milk; thus, future work normalizing the relative abundances of taxa based on the total bacterial load would provide more detailed information on how microbial communities are changing. It is also important to note that there is a limited number of samples of gangrenous mastitis due to the complexity of collecting this material because of the high deterioration of udder tissues and little available milk, which may restrict a more accurate analysis of beta diversity. Thereby, the results of this analysis should be interpreted with care. Furthermore, it is important to recognize that 16S rRNA gene sequencing captures the taxonomy and generate possible functional profiles, not the real function, of the microbial communities present. Although we aim to predict microbial metagenomes, future studies replicating this work should use shotgun metagenomic sequencing to allow a more in-depth analysis of microbial genes and their functions in goat milk, in different situations and types of goat mastitis.

## Conclusion

Here, we demonstrate the implications that occur in the milk microbiota in goat mammary glands when animals are affected by three different types of mastitis: subclinical, clinical, and gangrenous mastitis. We also highlight the main bacteria constituting the microbiota in the milk of healthy animals. With more severe mastitis, the richness of the microbiota is reduced; in gangrenous mastitis, the microbiota almost disappears. We highlight a new association of microorganisms in gangrenous mastitis in goats, with the agents *Escherichia* sp./*Shigella* sp. and *Enterococcus* sp. We identified important agents among the types of mastitis and healthy animals, such as *Bifidobacterium* sp., *Prevotella* sp., Ruminococcaceae family, *Providencia* sp., *Alkalibacterium*, and *Mycoplasma* sp. Furthermore, we report the importance of the L-tryptophan biosynthesis pathway and the sucrose degradation III (sucrose invertase) pathway in the prediction of the functional metabolite profile of the microbiota among the groups studied. Finally, the presence or absence of various new and known pathogenic genera in goat milk can be of paramount importance for the veterinary pharmaceutical industry and for the processing of dairy products.

## Data availability statement

The DNA sequences generated and analyzed during the current study are available in the NCBI SRA repository under BioProject PRJNA836133. Other data from the study are available from the corresponding author upon reasonable request.

## Ethics statement

The experimental protocol was approved by the Ethics Committee (Comissão de ética no uso de animais – CEUA) of the Federal University of Viçosa (UFV), according to protocol number 43/2016.

## Author contributions

RP conceived the study, conducted the experiment, and wrote the manuscript. RP, PV, TM, RY, LS, and JF analyzed the data. RP, MC, and MM reviewed the manuscript. All authors contributed to the article and approved the submitted version.

## Funding

This study was supported by the Coordenação de Aperfeiçoamento de Pessoal de Nível Superior—Brasil (CAPES) – Finance Code 001, the CNPq (Conselho Nacional de Desenvolvimento Científico e Tecnológico, Brasília, Brazil) and the FAPEMIG (Fundação de Amparo à Pesquisa de Minas Gerais, Belo Horizonte, Brazil). MM is supported by CNPq.

## Conflict of interest

The authors declare that the research was conducted in the absence of any commercial or financial relationships that could be construed as a potential conflict of interest.

## Publisher’s note

All claims expressed in this article are solely those of the authors and do not necessarily represent those of their affiliated organizations, or those of the publisher, the editors and the reviewers. Any product that may be evaluated in this article, or claim that may be made by its manufacturer, is not guaranteed or endorsed by the publisher.

## Footnotes

^1^ https://github.com/yhwu/idemp

^2^ https://benjjneb.github.io/dada2/tutorial.html

^3^ https://github.com/guokai8/microbial
