# Supplementary material for: Distinguishing the milk microbiota of healthy goats and goats diagnosed with subclinical mastitis, clinical mastitis, and gangrenous mastitis
Source: Front Microbiol. 2022 Aug 25;13:918706. doi: 10.3389/fmicb.2022.918706 (PMC9453028; doi:10.3389/fmicb.2022.918706)
Supplement: Supplementary file 1 [file Table_1.docx]

**Supplementary File 1**

**Supplementary Table S1 -** Collection sites and quantity of milk samples by types of goat mastitis in the microregions of the Zona da Mata of Minas Gerais, Brazil.

| MICROREGIONS FOR SAMPLE COLLECTION | | | | | | | |
| --- | --- | --- | --- | --- | --- | --- | --- |
| Clinical status | **Total Samples** | **Cataguases -Minas Gerais - Brazil** | **Juiz de Fora -Minas Gerais - Brazil** | **Manhuaçu-Minas Gerais - Brazil** | **Muriaé-Minas Gerais - Brazil** | **Ubá-Minas Gerais - Brazil** | **Viçosa-Minas Gerais - Brazil** |
| Clinically healthy | 12 | 0 | 3 | 3 | 0 | 3 | 3 |
| Subclinical mastitis | 42 | 4 | 12 | 5 | 8 | 4 | 9 |
| Clinical mastitis | 16 | 0 | 2 | 1 | 0 | 3 | 10 |
| Gangrenous Mastitis | 2 | 0 | 0 | 0 | 0 | 0 | 2 |

**Supplementary Table S2 -** Alpha Diversity Analysis - significance test of ANOVA for Shamnnon and Cha01 índices.

| ANOVA | | | | | | |
| --- | --- | --- | --- | --- | --- | --- |
| Summary(aov.Shannon) | Df | Sum-Sq | Mean-Sq | F-value | Pr(>F) |  |
| Types_of_Mastitis | 3 | 17.57 | 5.856 | 6.83 | 0.000432 | *** |
| Residuals | 68 | 58.30 | 0.857 |  |  |  |
| Summary (aov.Chao1) | Df | Sum-Sq | Mean-Sq | F-value | Pr(>F) |  |
| Types_of_Mastitis | 3 | 23571 | 7857 | 5.651 | 0.00162 | ** |
| Residuals | 68 | 94543 | 1390 |  |  |  |

Signif. codes: |0=‘***’| - |0.001=‘**’| - |0.01=‘*’| - |0.05=‘.’| - |0.1=‘’ | - |1|

**Supplementary Table S3 -** Alpha Diversity Analysis for index Shannon- Tukey’s honest significance test of our ANOVA - Tukey multiple comparisons of means - 95% family-wise confidence level.

| Shannon | | | | | |
| --- | --- | --- | --- | --- | --- |
| Types_of_Mastitis | diff | lwr | upr | p | adj |
| M1 vs H0 | -0.1151467 | -0.913354 | 0.68306053 | 0.9811726 |  |
| M2 vs H0 | -0.8164611 | -1.747703 | 0.11478075 | 0.1060118 |  |
| M4 vs H0 | -2.6256268 | -4.488110 | -0.76314314 | 0.0022984 | * |
| M2 vs M1 | -0.7013143 | -1.417727 | 0.01509862 | 0.0572259 |  |
| M4 vs M1 | 2.5104800 | -4.275383 | -0.74557735 | 0.0020641 | * |
| M4 vs M2 | -1.8091657 | -3.638088 | 0.01975692 | 0.0535992 |  |

Signif. codes: |0=‘***’| - |0.001=‘**’| - |0.01=‘*’| - |0.05=‘.’| - |0.1=‘’ | - |1|

**Supplementary Table S4 -** Analysis of Alfa Diversity for index Chao1- Tukey's honest significance test of our ANOVA - Tukey multiple comparisons of means - 95% family-wise confidence level

| Chao1 | | | | | |
| --- | --- | --- | --- | --- | --- |
| Types_of_Mastitis | diff | lwr | upr | p | adj |
| M1 vs H0 | -9.214286 | -41.35916 | 22.930587 | 0.8742375 |  |
| M2 vs H0 | -32.208333 | -69.71068 | 5.294018 | 0.1173179 |  |
| M4 vs H0 | -100.833333 | -175.83804 | -25.828631 | 0.0039569 | * |
| M2 vs M1 | -22.994048 | -51.84495 | 5.856858 | 0.1637950 |  |
| M4 vs M1 | -91.619048 | -162.69404 | -20.544060 | 0.0061787 | * |
| M4 vs M2 | -68.625000 | -142.27816 | 5.028156 | 0.0766860 |  |

Signif. codes: |0=‘***’| - |0.001=‘**’| - |0.01=‘*’| - |0.05=‘.’| - |0.1=‘’ | - |1|

**Supplementary Table S5** - Statistical analysis of beta diversity. Pairwise Permanova, considering the Bray method in the calculation, and with the Bonferroni correction.

| Beta Diversity | | | | | | | | |
| --- | --- | --- | --- | --- | --- | --- | --- | --- |
| Types_of_Mastitis | Df | SumsOfSqs | F,Model | R2 | p_value | sig | p_adjusted | sig |
| H0 vs M1 | 1 | 0,19159 | 1,02142 | 0,01926 | 0,34800 |  | 1,000 |  |
| H0 vs M2 | 1 | 0,53994 | 1,77689 | 0,06397 | 0,04900 | ‘.’ | 0,294 |  |
| H0 vs M4 | 1 | 0,73419 | 2,99713 | 0,19985 | 0,01300 | ‘*’ | 0,078 | ‘’ |
| M1 vs M2 | 1 | 0,98326 | 4,31004 | 0,07146 | 0,00100 | ** | 0,006 | ‘*’ |
| M1 vs M4 | 1 | 0,89803 | 4,82667 | 0,10308 | 0,00500 | ** | 0,030 | ‘’ |
| M2 vs M4 | 1 | 0,53633 | 1,43955 | 0,08255 | 0,10900 | ‘ ’ | 0,654 |  |

Signif. codes: |0=‘***’| - |0.001=‘**’| - |0.01=‘*’| - |0.05=‘.’| - |0.1=‘’ | - |1|
