# Supplementary material for: Distinguishing the milk microbiota of healthy goats and goats diagnosed with subclinical mastitis, clinical mastitis, and gangrenous mastitis
Source: Front Microbiol. 2022 Aug 25;13:918706. doi: 10.3389/fmicb.2022.918706 (PMC9453028; doi:10.3389/fmicb.2022.918706)
Supplement: Supplementary file 4 [file Image_1.pdf]

Supplementary Figure S1.

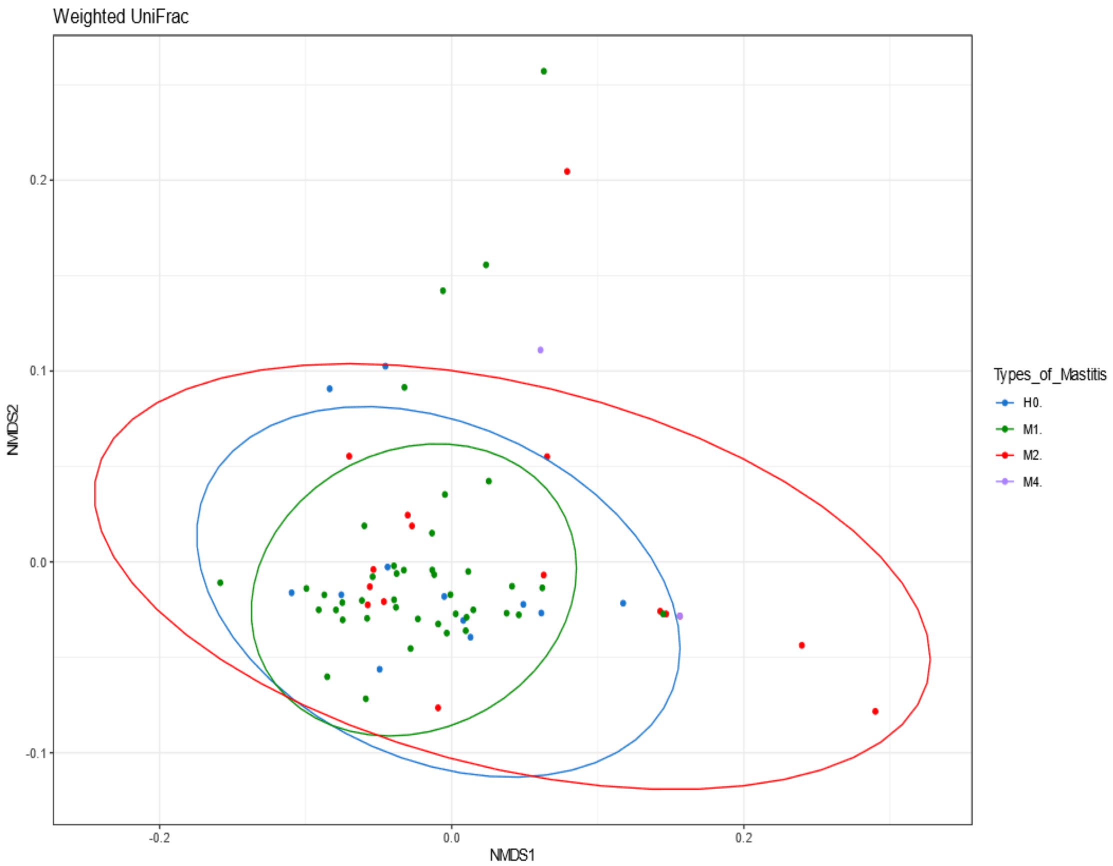

Unifrac weighted distance based NMDS plot explains the phylogenetic relationship between species for beta diversity with respect to healthy (H0), subclinical mastitis (M1), clinical mastitis (M2) and gangrenous mastitis (M4) groups. Each color depicts a different group or type of mastitis. The UniFrac weighted distance uses species abundance information and weights the branch length with the difference in abundance.
