# Supplementary material for: Distinguishing the milk microbiota of healthy goats and goats diagnosed with subclinical mastitis, clinical mastitis, and gangrenous mastitis
Source: Front Microbiol. 2022 Aug 25;13:918706. doi: 10.3389/fmicb.2022.918706 (PMC9453028; doi:10.3389/fmicb.2022.918706)
Supplement: Supplementary file 5 [file Image_2.pdf]

**Supplementary Figure S2.**

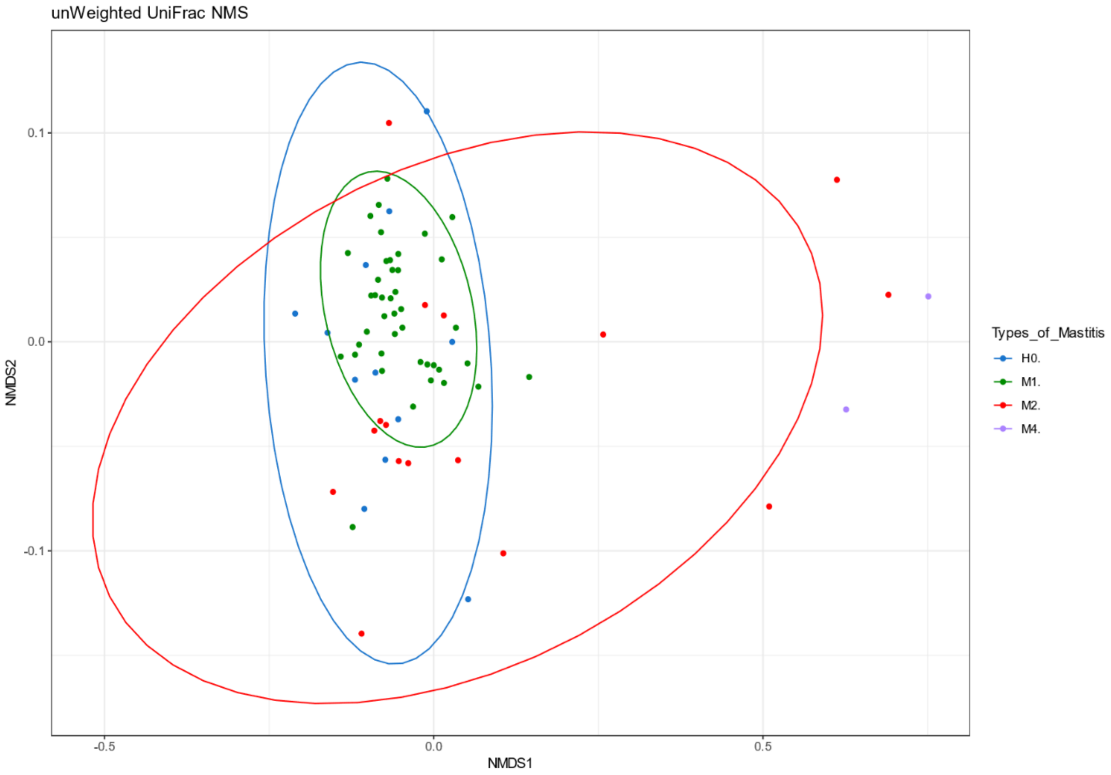


Unifrac unweighted distance based NMDS plot explains the phylogenetic relationship between species for beta diversity with respect to healthy (H0), subclinical mastitis (M1), clinical mastitis (M2) and gangrenous mastitis (M4) groups. Each color depicts a different group or type of mastitis. The UniFrac unweighted distance considers only the presence and absence of species information and counts the fraction of the length of the single branch for each community.
